# De novo ventricular tachycardia ablation with stacked pulsed-field and radiofrequency energy via a dual-energy lattice-tipped catheter

**DOI:** 10.1016/j.hrcr.2025.01.016

**Published:** 2025-01-31

**Authors:** James Mannion, Jonathan Lyne

**Affiliations:** 1 Electrophysiology Department, Beacon Hospital, Sandyford, Dublin 18, Ireland; 2 Cardiology Department, Cork University Hospital, Wilton, Cork, Ireland; 3 School of Medicine, University College Dublin, Belfield, Dublin 4, Ireland

**Keywords:** Combined stacked energy ablation, Pulsed field ablation, Radiofrequency energy, Sphere-9 catheter, Ventricular tachycardia ablation

**Key Teaching Points**

• Pulsed field energy can be successfully applied to acute RF lesions in the ventricle without complications.
• Combining RF and PF energy in the ablation of VT may allow for individualized ablation strategies depending on the target substrate/location, combining the strengths of each energy source.
• Despite only being licensed for atrial arrhythmias, a lattice-tipped dual-energy mapping and ablation catheter can successfully be used in de novo procedures to map and ablate VT in ischemic cardiomyopathy.

## Introduction

The role of pulsed field (PF) energy in ablation of ventricular tachycardia (VT) is not yet well established, with mixed short-term outcomes in initial clinical data as a single energy source, using mostly a standard tipped ablation catheter.^1^^,^^2^

PF shows potential superiority in terms of ventricular lesion size to radiofrequency (RF) in preclinical animal and computational models.^3^, ^4^, ^5^, ^6^ PF lesion dimensions increase with recurrent pulses via repetition dependence.^7^ PF energy also can be applied over preexisting chronic RF lesions.^8^ In these circumstances, PF demonstrates improved penetration through heterogenous or superficial fibrosis when compared with secondary superimposed RF.^8^

Ablation catheter technology also continues to advance, and compared with standard tipped catheters, a novel lattice-tipped ablation catheter has been found to generate larger lesion sizes during RF application.^3^ Currently licensed only for atrial ablation, this lattice-tipped dual-energy catheter has been used in the treatment of VT.^9^^,^^10^ In that study, patients who have already failed 1 RF VT ablation were selected, and a strategy of repeat ablation with RF as the primary energy source had promising results, with 78% of participants free from VT at 3 months.^10^ PF was generally reserved for patients who did not achieve acute procedural markers of success with RF.

In this case, we demonstrate the feasibility of using the dual-energy lattice-tipped catheter in de novo VT ablation for ischaemic cardiomyopathy, with stacked PF energy applied over acute RF lesions in areas of interest with good success.

## Case report

A 60-year-old man with ischaemic cardiomyopathy (LV ejection fraction ∼25% with inferior and inferolateral akinesis) and a cardiac resynchronisation therapy defibrillator in situ, presented with recurrent episodes of presyncope and collapse. His symptoms were highly debilitating with no association to exertion or positional change, lasting several minutes at a time. When his device was interrogated, we identified 51 runs of VT within the last 3 months, mostly self-limiting; however, some episodes required antitachycardia pacing therapy. He did not report any recent chest pain, and high sensitivity troponin was not elevated. No reversible predisposing factors were identified. There were no clinical or echocardiographic features of volume overload.

Medications included aspirin 75 mg once daily (OD), atorvastatin 80 mg OD, sacubitril/valsartan 24/26 mg twice daily, bisoprolol 5 mg OD, dapagliflozin 10 mg OD, spironolactone 12.5 mg OD, isosorbide mononitrate 60 mg OD, pantoprazole 40 mg OD, venlafaxine 150 mg OD, and dutasteride 500 μg OD.

**Figure 1:**
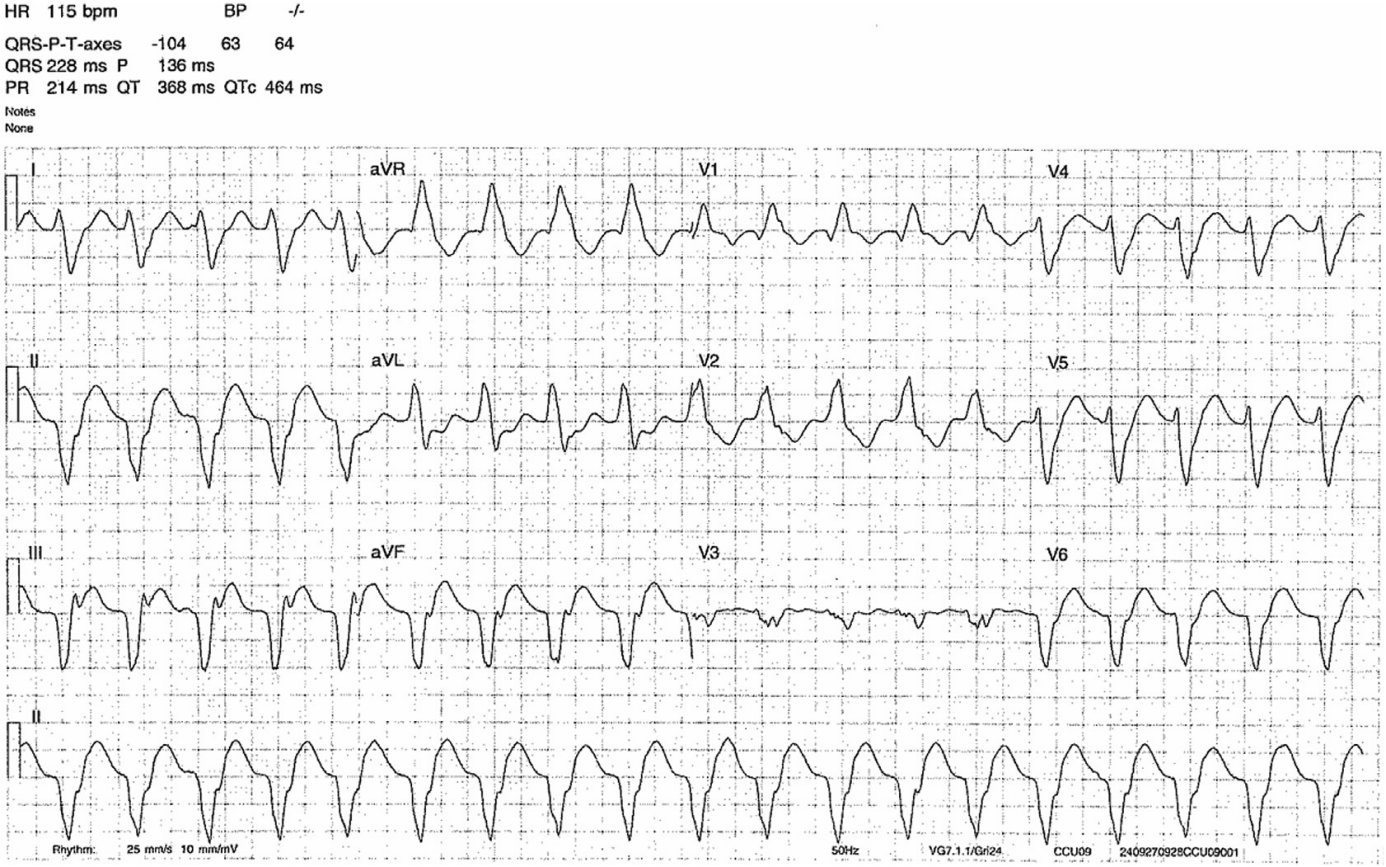
Clinical monomorphic ventricular tachycardia (VT), with exit site in the mid inferior wall of the left ventricle, in keeping with previous ischemia.

He received intravenous amiodarone loading therapy, but experienced further uncontrolled symptomatic monomorphic VT, at a rate of 115 beats/min (Figure 1), with the likely VT exit site in the mid-inferior wall of the left ventricle (LV). We discussed treatment options, and it was agreed to undertake a VT ablation with dual energy sources. Informed consent was obtained in the use of this catheter for the procedure.

The procedure was performed off of anti-arrhythmic therapy via a retrograde approach under general anesthetic, with heparin administration via activated clotting time guidance to a target of 300–350 milliseconds.

**Figure 2:**
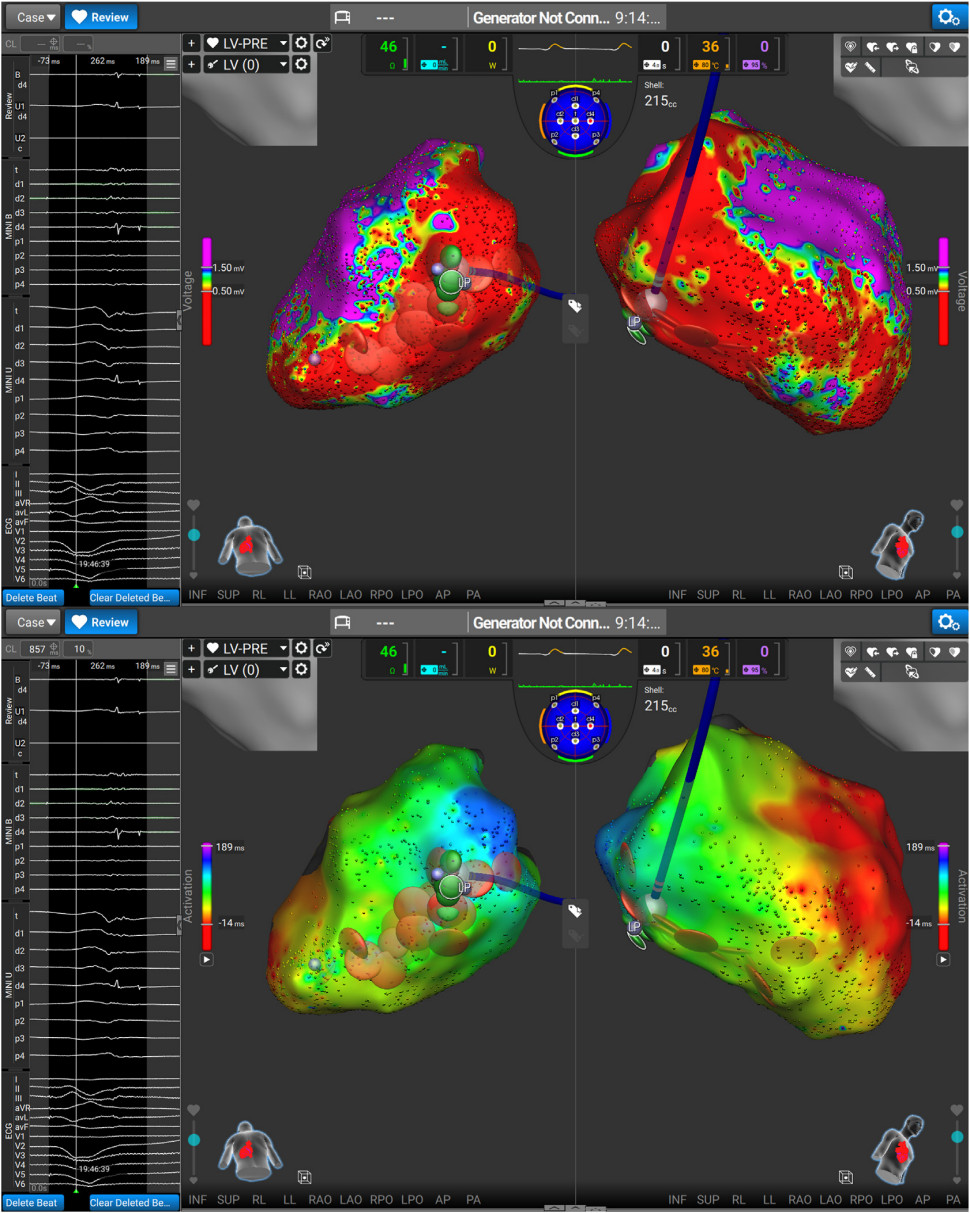
(Top) Bipolar voltage map of the ventricle, with large territory of scar (<0.5 mV) on the inferior wall. (Bottom) Local activation time map in sinus rhythm.

The LV was mapped with a dual-energy lattice tip mapping and ablation catheter (Sphere-9 Catheter, Affera Mapping and Ablation System, Medtronic Inc., Minneapolis, MN). Bipolar voltage mapping was performed with <0.5 mV as a low-voltage surrogate for LV fibrosis. Large areas of fibrosis were noted in the inferior/inferolateral walls (Figure 2).

**Figure 3:**
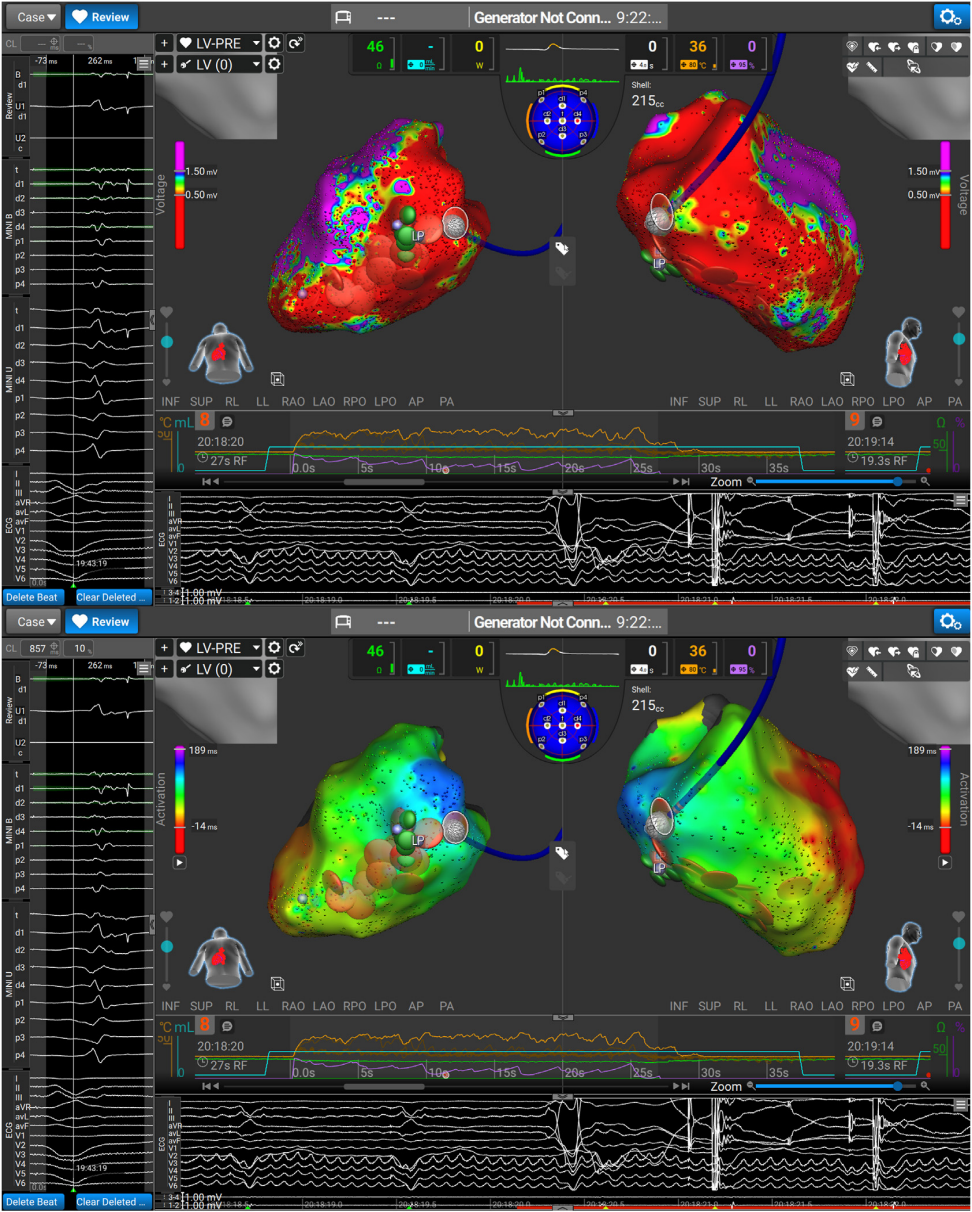
(Top) Areas of late potentials (LP) and fractionation during biventricular pacing, and the target for our dual-energy ablation. (Bottom) Local activation time map in sinus rhythm.

**Figure 4:**
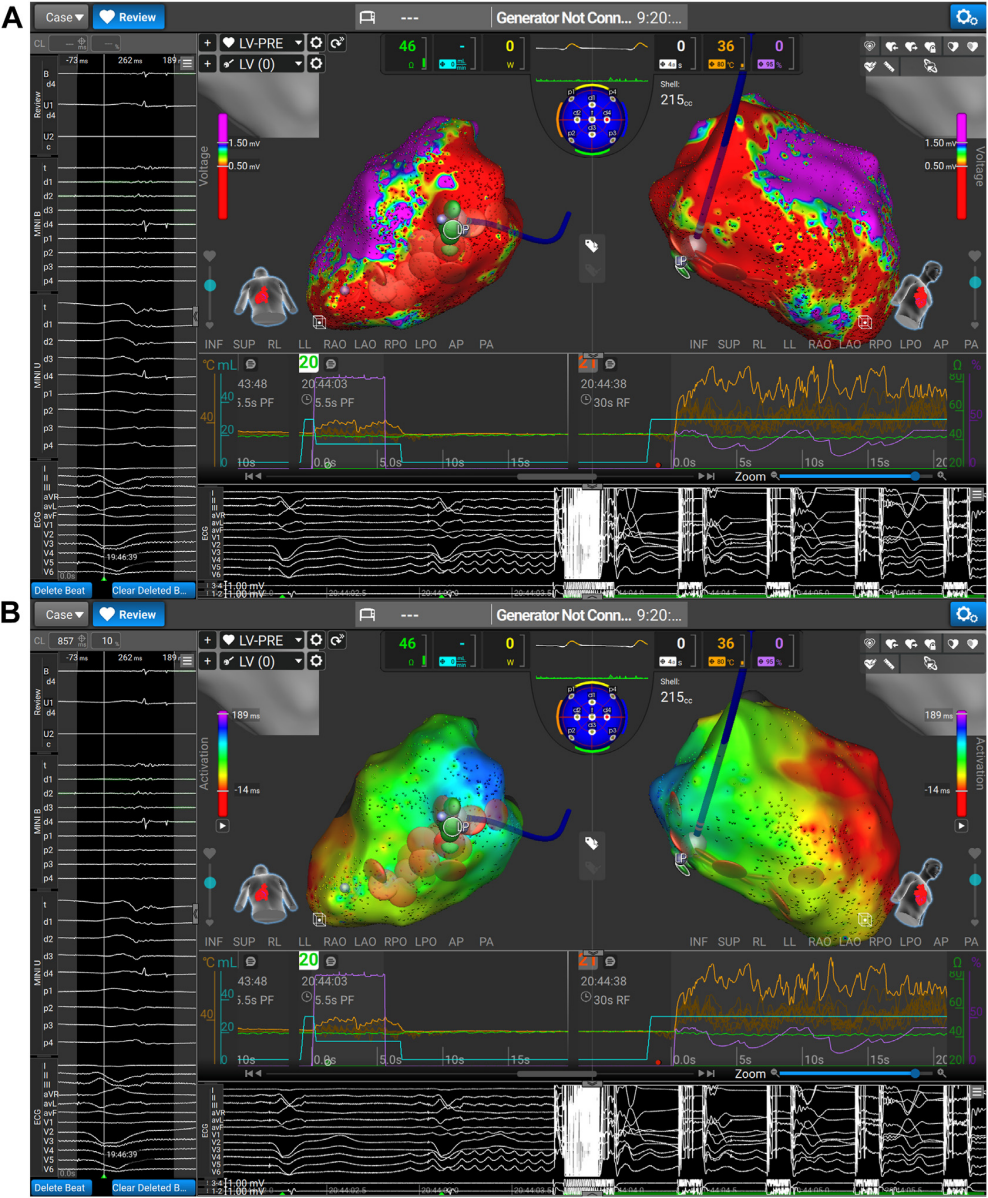
(Top) Radiofrequency (**A**) and subsequent pulsed-field energy (**B**) being applied to the areas of interest (Marked LP), with sequential applications compounding on each other. (Bottom) Local activation time map in sinus rhythm.

The clinical VT was readily inducible at the start of the case but was nonsustaining, so comprehensive tachycardia activation mapping or entrainment could not be performed despite several attempts. Instead, areas of fractionation and late activation during biventricular pacing were targeted, as demonstrated in Figure 3. RF was used in the first instance (Figure 4A) in 30- to 60-second aliquots, with a 40% current limit, aiming for a target temperature of 60 °C. When satisfactory RF lesions were completed, the energy source was toggled to PF, with further stacked applications over the RF as demonstrated in Figure 4B. The PF settings involved 1500 pulses, over 12 trains with a 350-millisecond inter-train delay. This inter-train delay results in a total application time of 5.5 seconds.

After ablation, the clinical VT could not be induced. There were no areas of late activation remaining in the scar region after the procedure, and the LV could not be captured by pacing over the areas of application. There were no acute complications, and postprocedural echo did not demonstrate any new effusion or LV dysfunction. There was no evidence of coronary spasm. The patient went to the intensive care unit for monitoring postprocedure. The device VT monitor zone was reduced to 110 to incorporate the rate of the previous clinical VT. On device interrogation 3 months after the procedure, the patient had remained free of any ventricular arrhythmia.

## Discussion

Currently RF is the most well-studied energy source in ablation involving the LV and remains the primary energy source of choice; however, innovations are necessary because RF is limited by arrhythmia recurrence.^11^ Ablation with RF as the only energy source may be limiting our lesion size, homogeneity, and resultant outcomes. PF has several proven preclinical advantages, with further theorized benefits from computational and animal models.^4^^,^^5^ PF has evidence of better penetration through areas of heterogenous or chronic superficial fibrosis; this theoretically lends well to the ablation of ventricular arrhythmia.^5^^,^^8^^,^^12^^,^^13^ Initial cohorts in which VT ablation is performed with PF as a single energy source, however, has yielded mixed results.^1^^,^^2^ Most of these data come from early generation standard-tipped ablation catheters, with a freedom from VT of 52% at 3 months by defibrillator interrogation.^1^ These energies penetrate and ablate tissue in different ways, and so combining them may be an answer to improve lesion quality.

This novel dual-energy lattice catheter currently has a Conformité Européenne certificate for the ablation of atrial arrhythmias only, but it has been validated for use in VT ablation.^9^^,^^10^ An initial case series of patients undergoing ventricular ablation with the dual-energy lattice-tip catheter (Sphere-9 Catheter, Affera Mapping and Ablation System, Medtronic Inc.) included 1 patient with ischaemic cardiomyopathy. The patient had RF and PF used in the procedure, and was free of VT up to the 5-month follow-up.^9^ In an observational study with 18 patients, the catheter was used with good success, using RF as the mainstay of therapy, with PF reserved for those in whom VT was not suppressed or achieved inadequate substrate modification.^10^ This study, however, only involved patients who already had a previous ablation. Overall this strategy achieved good success with freedom from VT acutely of 89% and up to 3 months of 78%.^10^

PF applications may be superimposed over chronic RF lesions very successfully, and this may modify substrate deep to the RF lesion though superficial fibrosis.^5^ Durable RF lesion application is the mainstay of therapy and so should be prioritized. We adhered to this application sequence for a number of reasons. Applying thermal RF to acutely ablated tissue after PF may theoretically lead to greater local thermal complications or limit possible RF application time. Furthermore, applying PF to already ablated tissue, this may be the reduced risk of ventricular capture/activation, which is a limitation of the energy source. Outcomes of ablations with RF as the sole energy source continue to be limited by VT recurrence^14^; however, using dual superimposed energy sources in this way may compound on the results of RF and further improve lesion homogeneity, depth, and size compared with RF alone. During application of PF in the LV, the standard atrial parameters of 1500 pulses, 12 trains over 4 seconds were adjusted, with an increased inter-train delay of 350 milliseconds, increasing the delivery time to 5.5 seconds. This technique was previously reported for the purpose of mitigating the risk of ventricular arrhythmia.^6^ Further research is likely needed to optimize parameters to ventricular tissue.

## Conclusion

Although the role of PF energy in ventricular ablation is not fully established, using both acute RF with superimposed PF applications is feasible in a de novo procedure. Dual-energy source use, with a focus on robust RF lesions first and supplemental PF, may result in increased lesion volume and homogeneity. Our patient demonstrated acute markers of procedural success, and there were no acute complications such as coronary spasm or ventricular capture. As we learn more about the behavior of PF in the ventricle, use of both energy sources may allow for individualized ablation strategies depending on target location and substrate characteristics.

## Disclosures

Prof J Lyne has received honoraria and speaking fees from Medtronic.
